# A New Challenge: Detection of Small-Scale Falling Rocks on Transportation Roads in Open-Pit Mines

**DOI:** 10.3390/s21103548

**Published:** 2021-05-19

**Authors:** Tiandong Shi, Deyun Zhong, Lin Bi

**Affiliations:** School of Resources and Safety Engineering, Central South University, Changsha 410083, China; 195511033@csu.edu.cn (T.S.); deyizhiyun@csu.edu.cn (D.Z.)

**Keywords:** intelligent mining, autonomous vehicle, obstacle detection, cloth simulation, lidar point cloud

## Abstract

In transportation at open-pit mines, rocks dropped as a mining truck is driven will wear out the tires of the vehicle, thus increasing the mining cost. In the case of autonomous vehicles, the vehicle must automatically detect rocks on the transportation roads during the driving process. This will be a new challenge: rough road, rocks of small size and irregular shape, long detection distance, etc. This paper presents a detection method based on light detection and ranging (lidar). It includes two stages: (1) using the modified cloth simulation method to filter out the ground points; (2) using the regional growth method based on grid division to cluster non-ground points. Experimental results show that the method can detect rocks with a size of 20–30 cm at a distance of 40 m in front of the vehicle, and it takes only 0.3 s on an ordinary personal computer (PC). This method is easy to understand, and it has fewer parameters to be adjusted. Therefore, it is a better method for detecting small, irregular obstacles on a low-speed, unstructured and rough road.

## 1. Introduction

Reducing the cost of transportation is an important goal for open-pit mines [1,2]. With the application of unmanned vehicles and light detection and ranging (lidar) equipment in a variety of enclosed scenarios [3,4,5], the transportation of open-pit mines is also expected to realize unmanned operations, but the literature [6,7,8] has proposed that the current unmanned vehicles will cause extra tire wear when facing bad road conditions. In addition, in the path planning task, the passability of rough roads needs to be analyzed [9,10,11,12]. Therefore, it is of great significance to realize the automatic detection of rocks on the road while the truck is driving.

At present, there is a lack of mature methods for detecting rocks on transportation road in open-pit mines. Although scholars in the field of autonomous driving have proposed a variety of obstacle detection methods [13,14,15,16], they are different from the main obstacles on transportation roads in mining. These methods deal with the detection of common objects on urban structured roads, such as motor vehicles, pedestrians, bicycles, etc. These objects are characterized by large scale, regular shape, and high recognition. Therefore, the problem of detecting rocks on transportation road in open-pit mines is a new challenge.

Obstacle detection methods based on lidar sensors can be divided into methods based on deep learning and traditional methods. Scholars such as Minemura [17] proposed an optimized convolutional neural network LMNet to handle point cloud target detection tasks, and expand the receptive field of the network by expanding convolution. 

This method runs fast, but sacrifices accuracy. Scholars such as Beltran [18] proposed a birdNet framework to process point cloud target detection tasks, projecting point cloud data into a cell code for bird’s-eye projection, and using a convolutional neural network to estimate the target position and direction. Scholars such as Abdelkarim [19] extended the yolo(you only look once) framework to the task of 3D point cloud target detection, which balanced the detection accuracy and speed. Scholars such as ShaoshuaiShi [20] proposed the PointRCNN framework, which divides the original point cloud into foreground and background, and directly generates a small amount of high-quality 3D region proposal from the point cloud in a bottom-up manner to perform accurate box refinement and confidence prediction. Deep learning-based methods require a large amount of labeled training data. The quality of the detection effect depends largely on the richness of the training data, and the principle is difficult to explain clearly.

Traditional methods generally assume that the road surface is locally flat. Scholars such as Tongtong Chen [21] proposed a ground segmentation method based on Gaussian process, using one-dimensional Gaussian process regression to classify the midpoint of each segment. This method divides the space into independent fan-shaped areas and cannot guarantee the continuity of the overall ground. Scholars such as Rummelhard [22] proposed a point cloud segmentation method based on spatio-temporal conditional random field, which considered the time sequence relationship between point cloud data. The estimated value of this method for each node is easily affected by its surrounding points. Scholars such as Thrun [23,24,25] proposed a height map method, which processed a 3D point cloud into a 2D raster map, and used methods such as height difference for classification within the raster. Although this method is highly efficient, its accuracy is difficult to adapt to unstructured roads. Scholars such as Narksri [26] proposed a ground segmentation method that is robust to slopes. This method deals with slopes on flat roads and cannot achieve satisfactory results for rough roads.

The above methods have been proved to be successful in some tasks, but the good results obtained by these methods are limited to the common obstacle types on structured roads. In the task of detecting small-scale and irregular-shaped obstacles on rough roads, it is difficult to obtain satisfactory results from these methods [21].

Transportation roads in open-pit mines are unstructured and rough, and the rocks on the roads are small and irregular in shape. These two characteristics require that the method of filtering ground points has strong generalization, and the accuracy of the method is high enough. This paper proposes a two-stage detection method for rock obstacles on transportation roads in open-pit mines. In the first stage, the cloth simulation method [27] in the field of computer graphics is introduced and modified to obtain an approximate simulation of the rough surface of the road and realize the filtering of ground points. The following modifications have been made to the original cloth simulation method: (1) limiting the movement of particles to the vertical direction. The original cloth simulation method is used to deal with three-dimensional simulation problems. The purpose of this paper is to obtain the degree of protrusion of the obstacle relative to the ground. The method only focuses on the z-axis coordinates of the point cloud. Therefore, the method limits the movement of particles in the z-axis direction. (2) The collision detection of particles is modified to compare the height difference. When the height of a particle is lower than the height of its adjacent grounding point, the particle needs to be set to be immovable, and its height must be reset to the height of its adjacent grounding point. This collision detection is intuitive and is a simple but very effective method that can simplify calculations and reduce time consumption. (3) The internal constraints between particles are simplified to the spring force between the current particle and the particles in its 4 neighborhoods. It will not have an obvious effect on the results, but it can reduce the time complexity of the method and reduce the running time of the program. This is a reasonable and effective modification. In the second stage, after filtering the ground points the non-ground points are clustered by grid division based on the regional growth method, and the bounding box of the object is calculated. The method proposed in this paper does not require the assumption of local flat of the road, and it is easy to understand.

The remainder of this paper is organized as follows. A new detection Algorithm 1 is proposed in Section 2. Section 3 presents the experimental results, and the proposed Algorithm 1 is discussed in Section 4. Finally, Section 5 concludes this paper.
**Algorithm 1.** Regional growth clustering based on grid division:
1: **Input:** 2: Point cloud = {P} 3: **Initialize:** 4: {grid_pts} ← {P}(project {P} to grid unit according to the coordinate) 5: {bin_mat} ← ∅ 6: {use_mat} ← ∅ 7: {label_mat} ← ∅ 8: Label ← 0 9: **Algorithm:** 10: ***for*** i = 1 to N step 1; j = 1 to M step 1 11:  ***if*** bin_mat[i][j]==0 or label_mat[i][j]≠0 12:  ***continue*** 13:  ***end if*** 14:  Creating a variable neighborPoints, which is used to store the neighborhood unit that meet the conditions. 15:  {neighborPoints}←∅ 16:  neighborPoints.push(grid_pts[i][j]) 17:  label + 1 18:  label_mat[i][j]←label 19:  ***while*** {neighboorPoints} is not empty 20:    cur_voxel←neighborPoints.pop() 21:    ***for*** k=1,2,3,4 22:      getting the index m,n of the k-th neighborhood grid unit of cur_voxel
23:      ***if*** bin_mat[m][n]==0 or use_mat[m][n]≠0 24:         ***continue*** 25:      ***end if*** 26:      label_mat[m][n]←label 27:       neighborPoints.push(grid_pts[m][n]) 28:       use_mat[m][n]←1 29:      ***end for*** 30:  ***end while*** 31:  ***end for*** 32: **Output:** 33:  {label_mat}

## 2. Method

**Figure 1:**
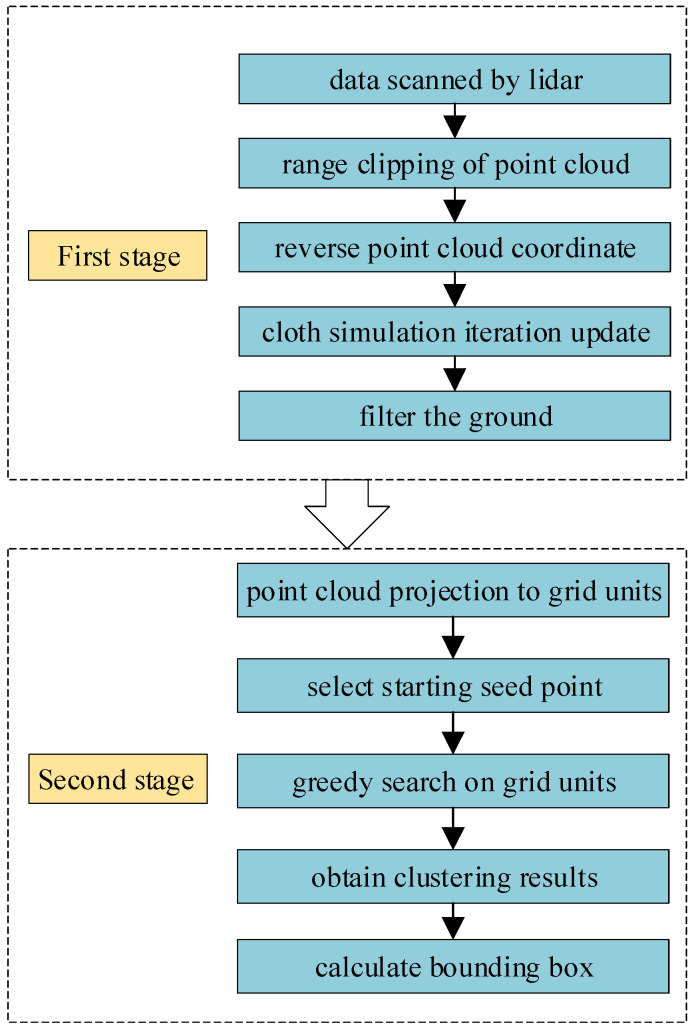
The main steps of the detection method based on cloth simulation and greedy search.

The method is divided into two stages. The first stage is to filter ground points. The original data are cropped and only the data in the valid range are retained. The z-axis coordinates of the point cloud are inverted and the modified cloth simulation method are used for ground recognition. The second stage is to cluster non-ground points. After filtering the ground points, the remaining points are projected into the grid unit according to its own coordinates. Setting a grid unit as the seed point, and the grid unit are clustered through the greedy search strategy. Finally, we calculate the bounding box for each clustered object. The code to implement this method is based on C++ and PCL(Point Cloud Library), and the technical route is shown in Figure 1.

### 2.1. Modification of Cloth Simulation Method 

**Figure 2:**
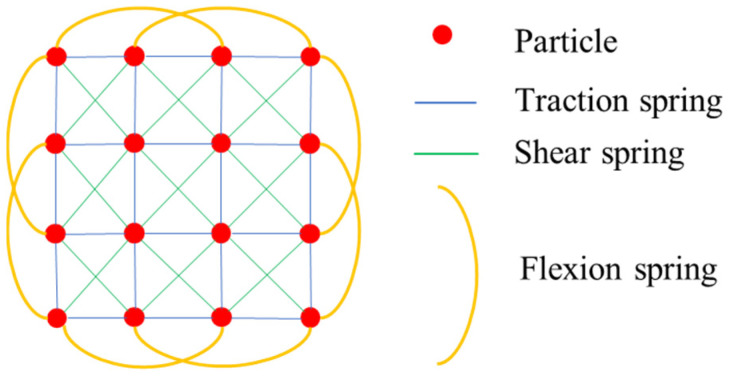
Cloth grid simulated in a computer program. Flexion springs resist bending stresses; sheer springs resist sheering stresses; traction springs resist stretching stresses.

Cloth simulation is a method that is used to simulate cloth in computer programs. In fact, the cloth in the real world is represented in a computer program as a grid connected by particles. The particles have mass and the interaction between the particles is called the mass-spring model [28], Figure 2 shows the structure of the grid. The particles represent the nodes of the grid. The nodes have no volume and are only given constant mass. Particles fall under the influence of gravity, and there is an interaction between connected particles during the falling process. The final position of the particles in space determines the final shape of the cloth.

**Figure 3:**
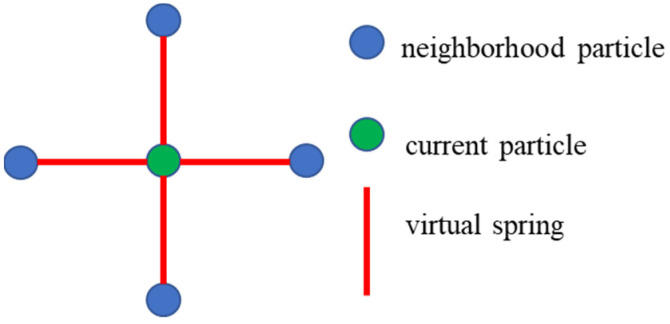
A representation of particle internal force constraints.

To solve the problem of filtering ground, the following modifications have been made to the cloth simulation method: (1) the movement of particles is simplified to one direction movement. The method only focuses on the z-axis coordinate information of the point cloud. When updating the position of the particles in each iteration, only the vertical position change of the particles needs to be updated. (2) The collision detection of particles is modified to height difference comparison. The movement of the particles is restricted to the vertical direction, so when the height of the particle is lower than the height of the adjacent ground point, the particle is set to be unmovable and the height of the particle is reset to the ground height. (3) The internal constraint between particles is expressed as the spring force between the current particle and its four neighboring particles. The purpose of this modification is to simplify the calculation steps without affecting the results and reduce the running time of the program. By calculating the distance between the current particle and the surrounding four neighboring particles, it is compared with the distance between the particles when the cloth is initialized. According to the amount of change between these two distances, the internal force of the particle can be calculated simply. Figure 3 shows the internal constraints between cloth particles.

In order to determine the shape of the cloth after each iteration, it is necessary to calculate the position of each particle after each iteration. The position of the particle is determined by the force on the particle. According to Newton’s Second Law, Formula (1) can be obtained:

(1) $$ma_{t}=F_{ext}+F_{int}$$

where $a_{t}$ represents the acceleration of the particle at time t; m represents the mass of the particle, which can be set as a constant; $F_{ext}$ represents the external force on the particle, usually referring to gravity, which can be expressed as a constant; $F_{int}$ represents the internal force on the particle. The vertical component of spring force is used as the internal force constraint on the current particle.

In this paper, the internal force between particles is expressed as a simple spring force model, calculated according to Formula (2):

(2) $$F_{int}=k\times \sum_{i=1}^{4}x_{i}\times sin\alpha_{i}$$

where k represents the coefficient of elasticity between particles, $x_{i}$ represents the change in the distance between particles during the falling process relative to the distance between particles when the cloth is initialized, and $sin\alpha_{i}$ represents the angle between the virtual spring and the horizontal plane.

The particle position after each iteration is obtained by the Taylor expansion method. We perform right Taylor expansion and left Taylor expansion on X(t), and omit the third term.

(3) $$X\left(t+\Delta t\right)=X\left(t\right)+V\left(t\right)\times \Delta t+a_{t}\times \frac{\Delta t^{2}}{2}$$

(4) $$X\left(t-\Delta t\right)=X\left(t\right)-V\left(t\right)\times \Delta t+a_{t}\times \frac{\Delta t^{2}}{2}$$

where V(t) represents the velocity of the particle at time t.

The particle position after each iteration is obtained by adding Formulas (3) and (4):

(5) $$X\left(t+\Delta t\right)=2X\left(t\right)-X\left(t-\Delta t\right)+a_{t}\times \Delta t^{2}$$

The relationship between the position of the particle and the force exerted by the particle is obtained simultaneously by Formulas (1) and (5):

(6) $$X\left(t+\Delta t\right)=2X\left(t\right)-X\left(t-\Delta t\right)+\frac{F}{m}\times \Delta t^{2}$$

where F represents the sum of external and internal forces experienced by the particle.

**Figure 4:**
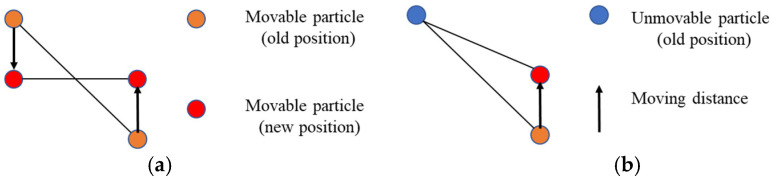
Schematic diagram of hole constraint. (**a**) Both particles can move; (**b**) only a single particle can move [29].

After inverting the original point cloud, small holes were formed by the bumps on the ground. Restricting the movement of particles in these holes [29] can speed up the convergence of the iterative falling process of particles. By comparing the height difference between the current particle and the surrounding particles, the particles of different heights are moved the same distance in the opposite direction. According to the different cloth hardness values, the particles can be moved multiple times to ensure faster convergence of the iteration process. The process diagram is shown in Figure 4. The moving distance can be calculated according to the following formula:

(7) $$d=\frac{1}{2}n\left(z_{i}-z_{0}\right)$$

where d represents the distance, each particle should move, n is 0 or 1, when the particle type is movable, n is 1, and when the particle type is unmovable, n is 0, and $z_{i}$ is the height value of the particle adjacent to the current particle, and $z_{0}$ represents the height value of the current particle.

### 2.2. Ground Filtering Based on Modified Cloth Simulation Method 

**Figure 5:**
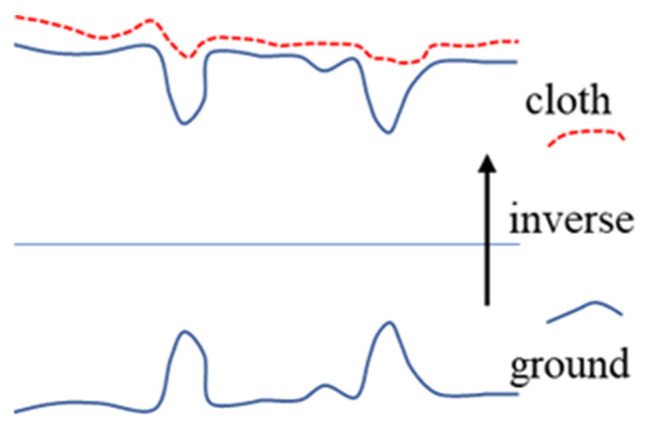
The idea of the cloth simulation method.

In order to accurately obtain the road surface from the point cloud, inspired by the cloth filtering method [29], we imagine a piece of cloth placed on the road surface and falling due to gravity. The final shape of the cloth is the shape of the ground containing obstacles. If the ground is reversed in advance, the final shape of the cloth is a pure road surface without obstacles. Based on this method, we have realized the filtering of rough ground, and achieved satisfactory results. Figure 5 shows the idea of this method.

First, we invert the z-axis coordinates of all points. Then, the cloth is simulated to fall under the constraints of external gravity and internal spring force. By affecting the interaction between particles and the iteration termination conditions, the final cloth shape can accurately represent the road surface. Finally, the cloth is used as a reference for classifying the original point cloud as ground points or non-ground points.

**Figure 6:**
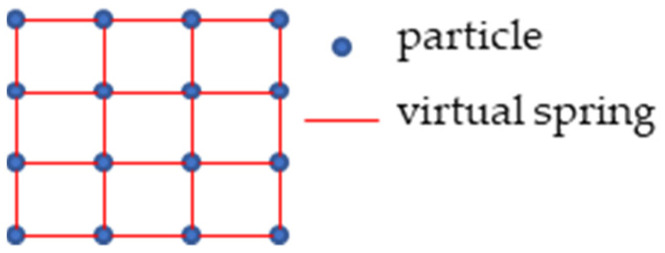
Cloth grid initialization.

The ground point recognition based on the cloth simulation method can be summarized as the following steps:

1. Inverting the original point cloud. Figure 5 shows the process.
2. Initiating the cloth grid and setting the height of all particles above the highest point of the point cloud. Figure 6 shows the process.
3. Projecting the grid particles and point cloud to the same horizontal plane, and finding the nearest ground point for each grid particle.
4. For each particle, calculating the displacement under the constraints of gravity and internal spring force. When the height of the particle is lower than the height of its nearest neighboring ground point, setting the particle as an unmovable particle.
5. For the particles in the hole region, calculating their displacement under the constraint of the height difference of the neighboring particles. Figure 4 shows the process.
6. Repeating 4–5, when the height change of all particles is small enough to reach the convergence condition, or the number of iterations exceeds the specified maximum number of iterations, the iteration process is terminated.
7. Comparing the distance between the points in the point cloud and the corresponding grid particles. Points smaller than the threshold are recognized as ground points; points greater than the threshold are recognized as non-ground points.

### 2.3. Grid Division Clustering Based on Regional Growth Method

After filtering ground points, non-ground points need to be clustered. The distribution of non-ground points in space is very discrete. At the same time, in order to speed up the search for neighboring points in space, this paper uses 2D grids to spatially divide the point cloud.

The core idea of the region growth method is to search from the seed point and merge the surrounding area with the target like the seed point according to the pre-defined rules, then forming a new seed point, and repeating the search process until there is no target that meets the conditions.

Before the greedy search, five variables need to be defined, which are the two-dimensional array grid_pts, bin_mat, use_mat, label_mat, and integer Label. Among them, grid_pts is used to describe the grid storing the point cloud, and the point cloud is projected to the corresponding grid unit according to their coordinates; bin_mat is used to indicate whether the current grid unit contains point cloud data, and its value is only 0 or 1; use_mat is used to indicate whether the current grid cell has been processed, and its value is only 0 or 1; label_mat is used to indicate which cluster the point cloud in the current grid cell belongs to; the integer Label is used to represent the total number of clusters.

First, the clustering algorithm needs to set the size of the grid, and project the points to be processed to the corresponding grid unit according to the coordinates. Cells that contain points inside are marked as real cells; cells that do not contain points inside are marked as empty cells. Then, the clustering algorithm uses a greedy search strategy to traverse and search all grid cells. The rule for judging whether a unit is added to the seed set is to judge whether the unit is a real unit and has not been processed. The pseudo code of the clustering algorithm is shown below.

After the clustering process, a bounding box needs to be calculated for each object. For each object after clustering, traversing all the points in the cluster, finding the coordinates of the points located on the boundary, and using them as the coordinates of the bounding box. For safety reasons, the bounding box can be appropriately enlarged. In other words, an expansion factor can be adopted to appropriately enlarge the bounding box.

### 2.4. Parameter Description

The method proposed in this paper has four parameters that need to be adjusted experimentally: the resolution of the cloth grid (grid resolution, GR); the height threshold (HT) that distinguishes ground points from non-ground points; the spring coefficient (SC); and the voxel resolution (VR) in the clustering process.

Due to the small volume of the falling rocks, there are few lidar points reflected by the rock itself, which is difficult to identify. This requires the resolution of the cloth grid to be set smaller. The height difference threshold depends on the size of the rocks, and it is necessary to ensure that the rocks are not mistakenly identified as ground points. The elastic coefficient between the cloth particles is determined by experiment. The grid resolution in the clustering process is related to the size of the rocks.

In addition to the above four parameters, there are three other parameters: the cloth hardness (CH), the max iteration times (MI), and the time step (TS) in the iteration process. But for all datasets, the values of these three parameters are the same. Through reference [29] and the experimentals, for all datasets, the cloth hardness value is set to 3, the max iteration times is set to 500, and the time step is set to to 0.65. These three parameters are set to these values to ensure that the program reaches the convergence condition.

## 3. Results

### 3.1. Data

**Figure 7:**
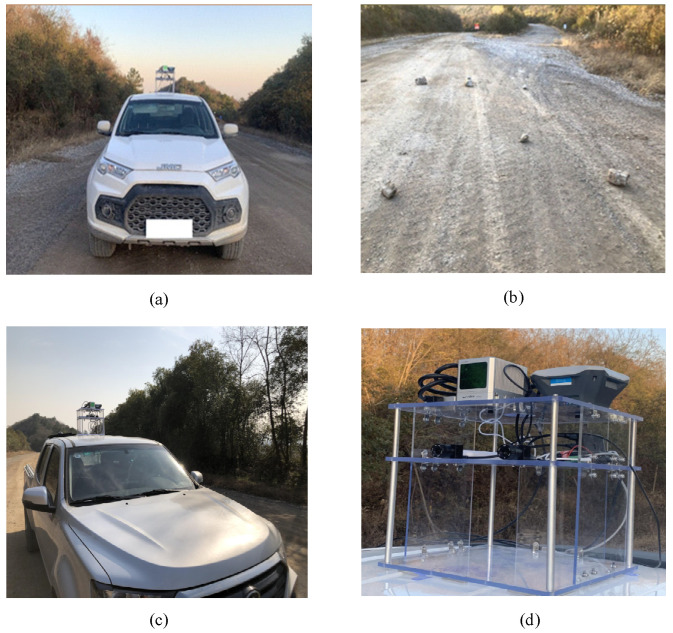
The place where the data was collected in the experiment. (**a**) Vehicles used in the experiment; (**b**) randomly placed rocks on the road; (**c**) side of the vehicle; (**d**) the detail of the equipment.

Data were collected on the transportation road of an open-pit mine. The equipment and rocks are shown in Figure 7. The lidar sensor used in the experiment was the DJI(Da Jiang Innovations) horizon. The horizon lidar uses non-repetitive scanning technology, and the area scanned by lidar will increase over time. The effective scanning distance with a reflectivity of 10% is 90 m, the horizontal field of view is 81.7°, and the vertical field of view is 25.1°. The horizon lidar is suitable for obstacle detection tasks on the road in front of the vehicle. The lidar sensor and other positioning equipment were installed on the top of the vehicle, and multiple rock obstacles were randomly placed on the road in front of the vehicle. The experiment measured four datasets at different distances. Table 1 shows the distance and rock size for each dataset.

Two things need to be explained about the data. First, the sizes of the rocks used in this paper were about 10–35 cm. Rocks of this size can cause damage to a tire and are difficult to detect by conventional methods. Those rocks larger than 40 cm in size are easily detected by conventional methods; rocks smaller than 10 cm in size will not cause damage to truck tires and do not need to be detected. Therefore, the rock size selected in this paper was representative. Second, the mining truck driver has a larger blind spot for observing the road ahead. The distance between the rocks and the vehicle in the experiment was greater than the effective observation distance of the mining truck driver. The above two points show that the size and distance of the rocks during the experiment had sufficient redundancy compared with the actual situation.

### 3.2. Result

According to the experimental results, for dataset2, dataset3, and dataset4, when the cloth grid resolution is set to 0.08 m, the height difference threshold for distinguishing ground points and non-ground points is set to 0.08 m, the coefficient of elasticity is set to 0.6, and the grid resolution in the clustering process is set to 0.5 m, the experimental results are good. The rocks on the road can be accurately detected and it does not take long. For dataset1, when the resolution of the cloth grid is set to 0.08 m, the threshold of the height difference between ground points and non-ground points is set to 0.05 m, the coefficient of elasticity is set to 0.8, and the grid resolution in the clustering process is set to 0.5 m, the experimental result is good. The best parameters of all datasets are shown in Table 2.

**Figure 8:**
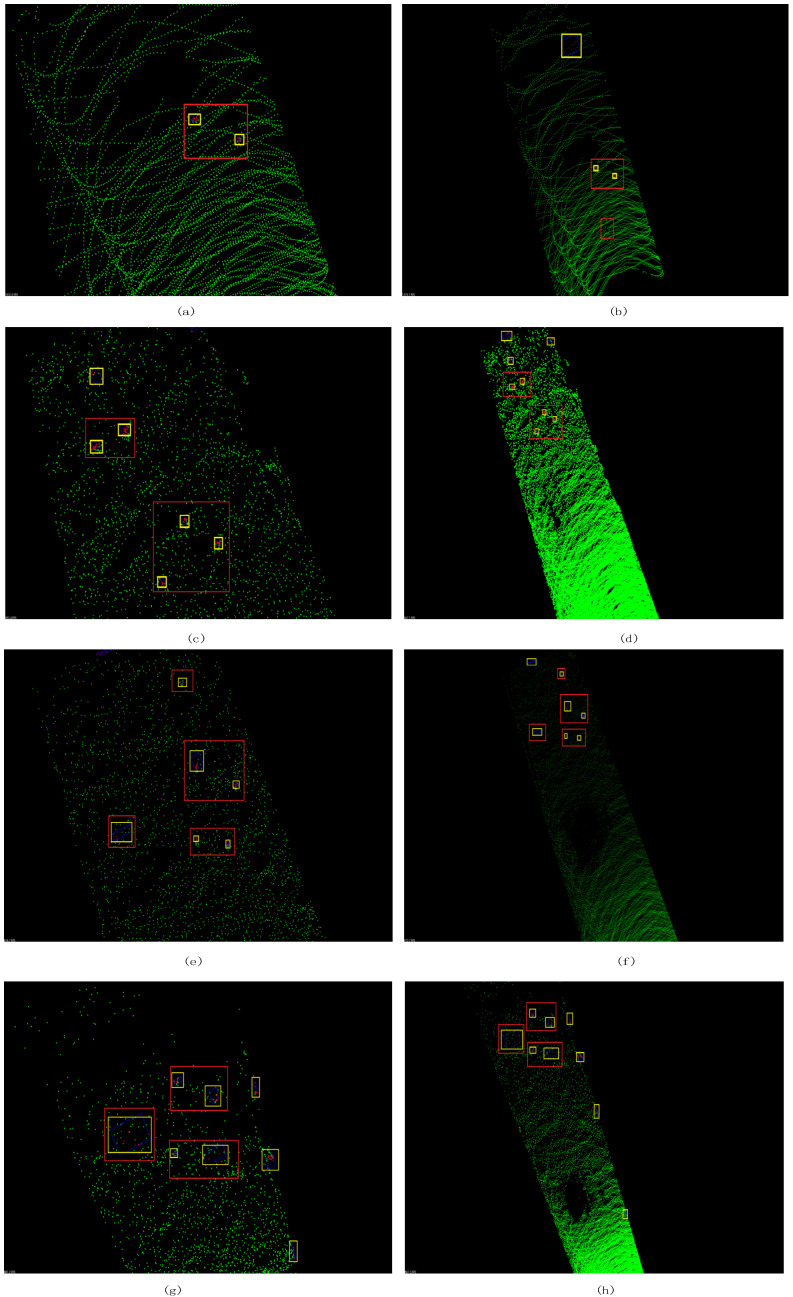
The experimental results of the whole dataset, the left is a partial enlarged view, and the right is a global view. The red rectangle includes the right detected rocks, the detected obstacles outside the red rectangle are errors (**a**,**b**) show the results of dataset1, two of the three rock blocks were detected; (**c**,**d**) show the results of dataset2, and five of the six rock blocks were detected; (**e**,**f**) show the results of dataset3, all six rock blocks have been detected; (**g**,**h**) show the results of dataset4, five of the six rock blocks were detected.

In this paper, qualitative analysis and quantitative analysis are used to evaluate the proposed method. For qualitative analysis, the detection results are visualized and compared with the original point cloud data. The visualization results of the four sets of data are shown in Figure 8.

In order to quantitatively analyze the results, this paper calculates two types of indicators on the data. The first type of index is the number of detected non-rocks objects divided by the total number of rocks. The lower the value, the better the experimental effect. The second type of index is the number of detected rocks divided by the total number of rocks. The higher the value, the more ideal the experimental effect. The calculation results are shown in Table 3.

**Figure 9:**
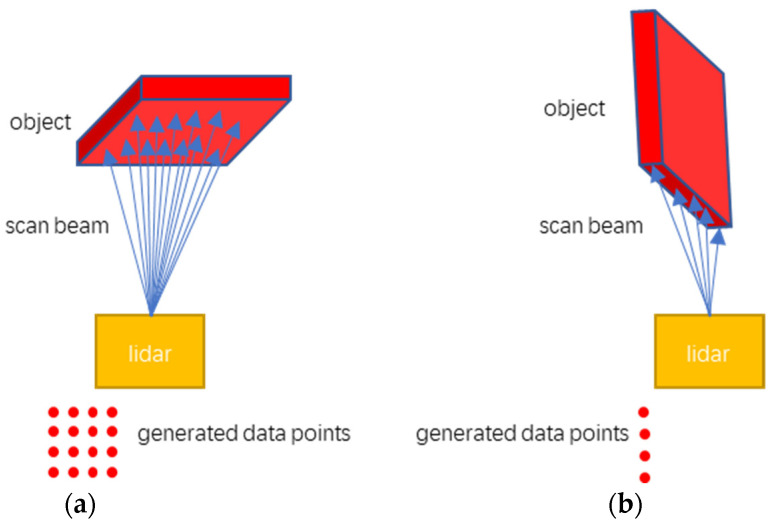
The relationship between object posture and point cloud density. Figure (**a**) shows the situation where the larger surface of the object points to the lidar; figure (**b**) shows the situation where the smaller surface of the object points to the lidar.

The red rectangle in Figure 8 includes the detected rocks. The detected obstacles outside the red rectangle are errors, which indicates that the method is wrong to identify the ground as an obstacle. It can be seen from the experimental results that for the third set of data, all six rocks were detected; but for the other three sets of data, some rocks were not detected. There are two reasons for this. One is that the effective scanning distance and accuracy of lidar equipment are limited. The lidar used in the experiment has an effective detection range of 90 m for a human-sized target. The size of the rocks used in the experiment is much smaller than the human target, so the lidar cannot scan small-sized rocks at a long distance. Another reason is the position and attitude of the rock relative to the lidar. The working principle of lidar is that the emitted light beam hits the surface of the object, and then the lidar receives the returning light beam, so the lidar can only scan a part of the entire surface of the object. When the surface area of the rock facing the lidar is very small, the reflected laser beam is very sparse, resulting in low density of the generated point cloud data. This effect is shown in Figure 9.

It can be seen from the experimental results that errors always occur at the farthest point of the point cloud or on both sides of the boundary. There are two reasons for the errors. One is that the detection accuracy of lidar at long distances has decreased. The lidar calculates the three-dimensional coordinates of the object based on the returned beam. For small-scale rocks at a long distance, the detection accuracy of the lidar decreases, so the calculated three-dimensional coordinates are distorted, which causes errors. Another reason is that the edge of the transportation road is very rough, so there are small chaotic rocks, which have an impact on the Algorithm 1.

## 4. Discussion

### 4.1. Result Analysis

The result is mainly affected by four factors, which are the resolution of the cloth grid, the threshold of the height difference between ground points and non-ground points, the elastic coefficient between cloth particles, and the grid resolution in the clustering process.

**Figure 10:**
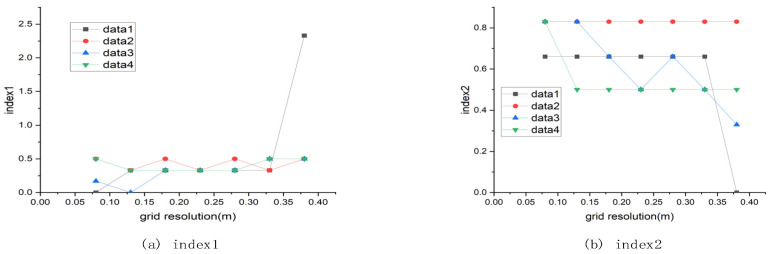
The effect of cloth mesh resolution on the results.

The higher the resolution of the cloth grid, the smaller the size of the grid and the greater the total number of particles. Considering the size of the rocks used in the experiment, the result is good when the cloth grid resolution is around 0.08 m after many experiments. The effect of different grid resolutions on the results is shown in Figure 10.

**Figure 11:**
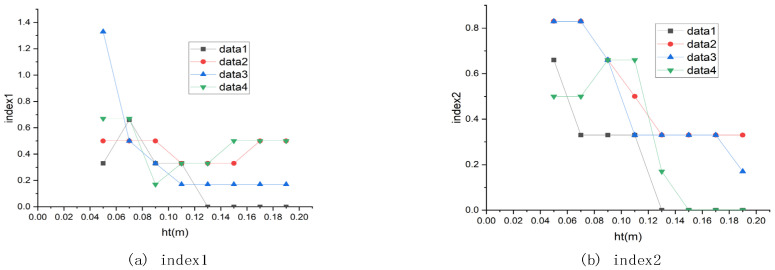
The effect of the height difference threshold on the results.

The height difference threshold that distinguishes ground points and non-ground points has an important influence on the results. After the iteration process is over, the final shape of the cloth grid represents the road surface. The height difference between the space point and its neighboring cloth particles is calculated and compared with the threshold. If the difference is less than the threshold, the space point is classified as a ground point; otherwise, the space point is classified as a non-ground point. If the height difference threshold is too large, the Algorithm 1 will incorrectly classify non-ground points as ground points; by contrast, if the height difference threshold is too small, the Algorithm 1 will incorrectly classify ground points as non-ground points. After many experiments, when the threshold value is around 0.08 m, the classification effect is good. The effect of different height difference threshold on the results is shown in Figure 11.

**Figure 12:**
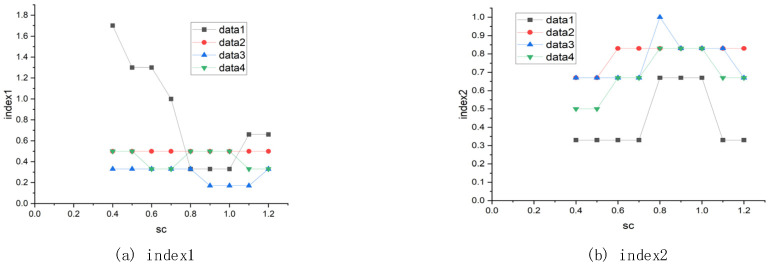
The effect of elasticity coefficient on the results.

The elastic coefficient between the cloth particles affects the internal spring force constraint. The function of the internal spring force constraint is to exert an upward force on the falling particles, which has an upward effect on the particles returning them to their correct positions. Considering an extreme case, if there is no spring force between the particles, the falling of the particles in the holes is only restricted by collision detection. The particles fall to a very deep position in the holes, which makes it impossible to accurately simulate the road surface. For different cloth grid resolution and height difference threshold, the best elastic coefficient can be determined through experiments. The effect of different elastic coefficients on the results is shown in Figure 12.

**Figure 13:**
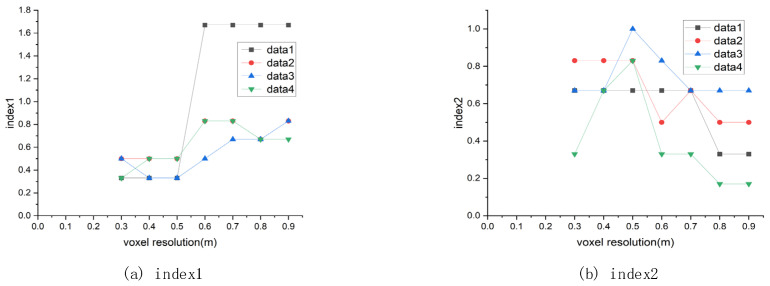
The effect of clustering grid resolution on the results.

The grid resolution in the clustering process affects the result of the Algorithm 1 for clustering non-ground points belonging to different objects. In the clustering process, the neighborhood of the seed point is searched. This search method has a great relationship with the grid size. When the Algorithm 1 searches for the neighborhood, the points belonging to different objects will be merged. The grid resolution for spatial division of the point cloud during the clustering process also needs to be carefully set. In the clustering process, if the size of the grid resolution is set too large, then multiple adjacent objects will be merged, which will cause the area of the bounding box to expand and reduce the area of the road where the vehicle can travel. After many experiments, it is finally determined that the grid resolution is 0.5 m, and satisfactory results are obtained for all datasets. The effect of different grid resolutions on the results is shown in Figure 13.

### 4.2. Further Analysis

The purpose of this paper was to detect rocks with a size of about 10–40 cm on the transportation roads of open-pit mines. Because the volume of these rocks is relatively small, and there is no fixed shape feature, the rocks are not easy to detect using conventional methods. At the same time, the transportation roads of open-pit mines are rough and unstructured, which requires a certain generalization of the method. A two-stage method proposed in this paper can effectively detect these rocks.

The first stage of the method uses a modified cloth simulation method to filter ground points from the original point cloud data. First, by reversing the original point cloud data, the convex part of the transportation road is transformed into a concave part, and the pure surface is located at the relatively highest point in the point cloud. Secondly, particles of constant mass fall from a height and, under the action of internal constraints, the particles will eventually stay near their corresponding actual ground points. Finally, the final shape of the cloth grid is determined, and the ground points can be identified by comparing them with the original point cloud data. This method is suitable for processing unstructured roads. After further modification, it can be used for rough road recognition in the field scene, and it has a certain versatility.

The second stage of the method uses grid division clustering based on area growth method to obtain the bounding box of each rock. In order to speed up the clustering process, this paper does not directly use a single point as the basic search unit, but uses a spatial grid division method to divide the point cloud into regular-sized grids unit. Each grid unit is used as the search unit of the area growth method, and the search process uses a greedy search strategy. The key to the clustering process is not to mistakenly identify multiple adjacent objects as one object, which will increase the volume of the bounding box and cause the truck’s drivable area to become smaller.

The physical properties of the object itself (such as density, material) also affect the scanning effect of the lidar sensor. The wavelength of the light beam emitted by the lidar is fixed, so the reflectivity of the light beam to objects of different densities is different, which will affect the density of the point cloud data and the detection results. The influence of the density of the scanned object on the reflectivity of the laser beam is complex, involving knowledge of optics and physics. Due to the lack of corresponding equipment and background knowledge, this paper does not undertake further research on this subject.

In addition, the method only uses the Z-axis coordinate information of the point cloud, and does not consider the local geometric characteristics of the point cloud. Future research may consider the X-axis and Y-axis coordinates of the point cloud, and then assist in the detection from the perspective of the local features of the point cloud.

### 4.3. Time Analysis

The machine used in the experiment is an ordinary personal computer (PC, (CPU-Intel Corei7, RAM-16G, OS-Ubuntu16). Under the best parameters, each group of data is tested multiple times, the fastest and slowest running time are counted, and the average time is calculated. The results are shown in Table 4.

The source code of the Algorithm 1 proposed in this paper is written in C++ language and uses central processing unit (CPU) multi-thread acceleration. It can be seen from Table 4 that the running time of the method is about 0.3 s. When the truck is loading ore, the driving speed is about 10 m/s. The running time of the method satisfies the reaction and avoidance time of the mining truck, so the method has a certain safety redundancy. Future research can use the graphics processing unit (GPU) parallel computing method to optimize the code, so that the program running speed is further accelerated to meet higher real-time requirements.

## 5. Conclusions

This paper proposes a new method for the detection of rock obstacles on rough roads during the driving process of mine trucks. First, the method modifies the cloth simulation method to make it applicable to the ground point filtering task of rough roads; secondly, the method uses the regional growth clustering method of grid division to achieve rapid clustering. The running speed of the method meets the real-time requirements of autonomous vehicles. Experiments show that for the high-density point cloud data scanned by lidar, the method can accurately detect rocks with a size of 20–30 cm and above at about 40 m within a safe time of 0.3 s. For a fully loaded mining truck with a driving speed of 30 km/h, this is sufficient time for the vehicle to avoid obstacles. The method is easy to understand, and there are fewer parameters that need to be adjusted. With high-precision lidar, the method can detect small-scale rocks on rough transportation roads in open-pit mines. This method can be extended to the detection of small-scale irregularly shaped obstacles on unstructured rough roads.

The effect of this method mainly depends on four parameters, which are the resolution of the cloth grid, the threshold of the height difference between ground points and non-ground points, the elasticity coefficient between cloth particles, and the grid resolution in the clustering process. The optimal values of these four parameters can be determined through experiments.

The problem discussed is a brand new research task, so there is a lack of mature and reliable methods. The method proposed in this paper also requires a lot of scenarios and data to verify it. The internal constraints between particles are simply expressed as spring forces in the method. Such a representation method cannot accurately simulate the internal interaction between particles. In addition, the effect of the method is related to the quality of the point cloud, which places higher requirements on the detection accuracy of the lidar. In order to improve the effect of the method, in the future we will try to extract features based on the geometric information of the object to optimize the detection process.

## Figures and Tables

**Table 1:** Rock size and placement distance.

Distance/m	Rock1 (cm^3^)	Rock2 (cm^3^)	Rock3 (cm^3^)	Rock4 (cm^3^)	Rock5 (cm^3^)	Rock6 (cm^3^)
12–17	20 × 20 × 23	40 ×20 × 20	18 ×15 × 10	—	—	—
35–40	14 × 10 × 8	15 × 12 × 14	25 × 15 × 13	33 × 19 × 25	32 × 18 × 21	10 × 10 × 10
36–44	14 × 10 × 8	15 × 12 × 14	25 × 15 × 13	33 × 19 × 25	32 × 18 × 21	10 × 10 × 10
44–52	14 × 10 × 8	15 × 12 × 14	25 × 15 × 13	33 × 19 × 25	32 × 18 × 21	10 × 10 × 10

**Table 2:** Parameter description.

Dataset	Distance/m	Number of Rocks	Parameter Value			
			GR	HT	SC	VR
1	12–17	3	0.05	0.05	0.8	0.5
2	35–40	6	0.08	0.08	0.6	0.5
3	36–44	6	0.08	0.08	0.6	0.5
4	44–52	6	0.08	0.08	0.6	0.5

**Table 3:** Index calculation results of the four sets of data.

Dataset	Number of Rocks Detected	Number of Non-Rocks Detected	Index1	Index2
1	2	1	1/3	2/3
2	5	3	1/2	5/6
3	6	2	1/3	1
4	5	3	1/2	5/6

**Table 4:** Time analysis.

Group	Number of Experiments	Fastest Time/s	Slowest Time/s	Average Time/s
1	20	0.28	0.30	0.29
2	20	0.29	0.31	0.30
3	20	0.28	0.31	0.30
4	20	0.29	0.32	0.30

## Data Availability

Not applicable.

## References

[1] Widdifield L., Riggle R. (2016). The Big Picture: An Overview Approach to Surface Mining. Min. Eng..

[2] Corporation V. (2015). A new approach to building haul roads. Eng. Min. J..

[3] Zhang H., Wang J. (2016). Active Steering Actuator Fault Detection for an Automatically-Steered Electric Ground Vehicle. IEEE Trans. Veh. Technol..

[4] Farinelli A., Iocchi L., Nardi D. (2004). Multi-robot systems: A classification focused on coordination. IEEE Trans. Syst. Man Cybern. Part B (Cybern.).

[5] Yuan K., Yuan L., Fang L.X. (2007). Recent Researches and Development on Multi- Robot System. J. Autom..

[6] Ayres A. (2016). Considerations When Implementing Autonomous Haulage in Open Cut Mining. https://amcconsultants.com/experience/dd-considerations-autonomous-haulage-open-cut-mining/.

[7] Parreira J. (2013). An Interactive Simulation Model to Compare an Autonomous Haulage Truck System with a Manually-Operated System. Ph.D. Thesis.

[8] Kansake B.A. (2020). Frimpong, S. Analytical modelling of dump truck tire dynamic response to haul road surface excitations. Int. J. Min. Reclam. Environ..

[9] Ziyu Z., Lin B. (2020). A New Challenge: Path Planning for Autonomous Truck of Open-Pit Mines in the Last Transport Section. Appl. Sci..

[10] Papadakis P. (2013). Terrain traversability analysis methods for unmanned ground vehicles: A survey. Eng. Appl. Artif. Intell..

[11] Gennery D.B. (1999). Traversability analysis and path planning for a planetary rover. Auton. Robot..

[12] Sock J., Kim J., Min J., Kwak K. Probabilistic traversability map generation using 3D-LIDAR and camera. Proceedings of the 2016 IEEE International Conference on Robotics and Automation (ICRA).

[13] Asvadi A., Peixoto P., Nunes U. (2016). Two-stage static/dynamic environment modeling using voxel representation. Robot 2015: Second Iberian Robotics Conference.

[14] Asvadi A., Cristiano P., Peixoto P., Nunes U. (2016). 3D Lidar-Based Static and Moving Obstacle Detection in Driving Environments: An Approach Based on Voxels and Multi-Region Ground Planes. Robot. Auton. Syst..

[15] Dairi A., Harrou F., Senouci M., Sun Y. (2018). Unsupervised Obstacle Detection in Driving Environments Using Deep-Learning-Based Stereovision. Robot. Auton. Syst..

[16] Darms M.S., Rybski P.E., Baker C., Urmson C. (2009). Obstacle Detection and Tracking for the Urban Challenge. IEEE Trans. Intell. Transp. Syst..

[17] Minemura K., Liau H.F., Monrroy A., Kato S. Lmnet: Real-Time Multiclass Object Detection on Cpu Using 3D Lidar. Proceedings of the 2018 3rd Asia-Pacific Conference on Intelligent Robot Systems (ACIRS).

[18] Beltran J., Guindel C., Moreno F.M., Cruzado D., Garcia F., de la Escalera A. Birdnet: A 3D Object Detection Framework from Lidar Information. Proceedings of the 2018 21st International Conference on Intelligent Transportation Systems (ITSC).

[19] Ali W., Abdelkarim S., Zidan M., Zahran M., El Sallab A. YOLO3D: End-to-End Real-Time 3D Oriented Object Bounding Box Detection from LiDAR Point Cloud. Proceedings of the European Conference on Computer Vision (ECCV) Workshops.

[20] Shi S., Wang X., Li H. PointRCNN: 3D Object Proposal Generation and Detection from Point Cloud. Proceedings of the IEEE/CVF Conference on Computer Vision and Pattern Recognition.

[21] Chen T.T., Dai B., Wang R.L., Liu D.X. (2014). Gaussian-Process-Based Real-Time Ground Segmentation for Autonomous Land Vehicles. J. Intell. Robot. Syst..

[22] Rummelhard L., Paigwar A., Negre A., Laugier C. Ground Estimation and Point Cloud Segmentation using SpatioTemporal Conditional Random Field. Proceedings of the 2017 IEEE Intelligent Vehicles Symposium (IV).

[23] Thrun S., Montemerlo M., Dahlkamp H., Stavens D., Aron A., Diebel J., Fong P., Gale J., Halpenny M., Hoffmann G. (2006). Stanley: The robot that won the DARPA Grand Challenge. J. Field Robot..

[24] Montemerlo M., Becker J., Bhat S., Dahlkamp H., Dolgov D., Ettinger S., Haehnel D., Hilden T., Hoffmann G., Huhnke B. (2008). Junior: The stanford entry in the urban challenge. J. Field Robot..

[25] Urmson C., Anhalt J., Bagnell D., Baker C., Bittner R., Clark M.N., Dolan J., Duggins D., Galatali T., Geyer C. (2008). Autonomous driving in urban environments: Boss and the urban challenge. J. Field Robot..

[26] Narksri P., Takeuchi E., Ninomiya Y., Morales Y., Akai N., Kawaguchi N. A Slope-robust Cascaded Ground Segmentation in 3D Point Cloud for Autonomous Vehicles. Proceedings of the 2018 21st International Conference on intelligent transportation systems (ITSC).

[27] Weil J. (1986). The synthesis of cloth objects. ACM Siggraph Comput. Graph..

[28] Provot X. (1995). Deformation constraints in a mass-spring model to describe rigid cloth behaviour. Graphics Interface.

[29] Zhang W., Qi J., Wan P., Wang H., Xie D., Wang X., Yan G. (2016). An Easy-to-Use Airborne LiDAR Data Filtering Method Based on Cloth Simulation. Remote Sens..

